# Radiological staging clinical decision support model for rectal cancer lymph node metastasis detection on MRI

**DOI:** 10.1186/s12885-026-16175-z

**Published:** 2026-05-22

**Authors:** Benjamin Keel, Aaron Quyn, David Jayne, Samuel D. Relton

**Affiliations:** 1 University of Leeds, Leeds, UK; 2 Leeds Teaching Hospitals NHS Trust, Leeds, UK

**Keywords:** Lymph node metastasis, Rectal cancer, MRI, Variational autoencoder, Multiple instance learning, Deep learning

## Abstract

**Background:**

Accurate staging of lymph node metastasis (LNM) is crucial for personalising rectal cancer treatment. Lymph nodes (LNs) are the most common sites of rectal cancer metastasis, and malignant LNs are typically treated with neo-adjuvant radiotherapy or chemoradiotherapy (CRT) to reduce the chance of recurrence and distant metastasis after surgery. Radiological staging criteria, based on LN size, shape, and texture, are known to be subjective, and radiologists have an average diagnostic performance of 73% sensitivity and 74% specificity.

**Methods:**

This study develops a fully automatic and end-to-end pre-operative radiological staging model for rectal cancer LNM using artificial intelligence (AI) methods. The model combines automatic detection of lymph nodes on Magnetic Resonance Imaging (MRI) with multiple instance learning patient-level lymph node staging. Models were trained and evaluated on an in-house dataset provided by Leeds Teaching Hospitals NHS Trust (LTHT) including 458 patients with pre-operative MRI scans, patient clinical data, and post-operative pathological TNM staging. Accurate detection of the lymph nodes on MRI is achieved using nnU-Net, and the classification results compare different 3D feature encoders, investigating a trade-off between performance and interpretability.

**Results:**

The results demonstrate state-of-the-art performance with cross-validated metrics of 0.828 AUC, 86.6% sensitivity, and 72.4% specificity. Clinical validation study results show that the AI staging model performance exceeded three expert radiologists in predicting post-operative pathology with a 9% higher F1 score.

**Conclusions:**

This study provides strong evidence that an end-to-end AI model can significantly improve pre-operative staging of rectal cancer lymph node metastasis. The model outperformed expert radiologists and shows clear potential to enhance clinical decision making and improve patient outcomes.

## Introduction

Colorectal cancer is the third most common cancer and the second leading cause of cancer related death worldwide, with 1.9 million cases and 900,000 deaths in 2020. By 2040, the incidence is expected to increase to 3.2 and 1.6 million respectively [1]. In the UK, rectal cancer (RC) is the fifth most common cancer and it has a 5-year survival rate of 59.6% [2]. Accurate pre-operative lymph node metastasis (LNM) staging is crucial for RC treatment planning, as LNM increases the risk of post-operative local recurrence and distant metastasis [3, 4].

This study presents a fully automatic and end-to-end artificial intelligence (AI) system to support pre-operative rectal cancer (RC) lymph node metastasis (LNM) staging on MRI. The segmentation model nnU-Net [5] was used to automatically detect lymph nodes (LNs) in the scans, providing precise border definitions which were used to crop to the region of interest LN MRI patches and to calculate size, shape and texture features. The classification model pipeline combines a 3D feature encoder model backbone with a multiple instance learning (MIL) multi-layer perceptron (MLP) classification architecture, which integrates the clinical and imaging data. The main experiment was to compare the performance and interpretability of different deep learning 3D feature extraction models, including Convolutional Neural Networks (CNN), Variational Autoencoders (VAE) and Vector Quantised Variational Autoencoders (VQ-VAE). Models were trained and evaluated on an in-house dataset provided by Leeds Teaching Hospitals NHS Trust (LTHT), consisting of 458 patients with pre-operative MRI scans, patient clinical data, and post-operative pathological TNM staging. The best performing model was validated with three expert radiologists.

According to the National Institute for Health and Care Excellence (NICE) guidelines [6], patients with suspicious lymph nodes (LNs) detected on MRI are generally considered for neo-adjuvant radiotherapy or chemoradiotherapy (CRT) as standard in clinical practice. CRT targets the primary tumour and regional LNs, and several clinical trials have shown that patients receiving CRT have less local recurrence with better overall and disease-free survival [6]. Radiological staging criteria for lymph nodes on MRI have 73% sensitivity [95% CI: 70-76%] and 75% specificity [95% CI: 73-77%] for pre-treatment staging of RC [7], leading to suboptimal treatment decisions and patient outcomes.

Recent studies have demonstrated the capability of deep learning methods for accurately staging RC LNM on pre-operative radiologic imaging, outperforming radiologists and methods using radiomics features, however, no previous studies have included patients with and without radiotherapy or chemoradiotherapy (CRT) in the same patient cohort [8].

To our knowledge, this is the first study to:

- Build a RC LNM staging model on MRI using a broadly inclusive patient cohort, combining patients with or without pre-operative CRT treatment to enhance generalisability across different clinical scenarios.
- Demonstrate that an AI staging model has the potential to outperform radiologists in diagnostic accuracy based on a clinical validation study of patients with and without CRT treatment.

## Materials and methods

### Dataset

This retrospective study included RC patients who underwent surgery at Leeds Teaching Hospitals NHS Trust (LTHT) between January 2010 and December 2022. The study cohort included 458 patients with a pre-operative T2-weighted MRI scan within 18 weeks of a total mesorectal excision (TME) surgery and post-operative pathological TNM staging available. T2-weighted MRI was selected because it provides the highest level of detail and is more sensitive to differences in water content than T1-weighted sequences, resulting in greater contrast in the soft tissues where the lymph nodes are located. The key outcome data is the pathological N-stage, where N0 represents no evidence of lymph node metastasis, N1 for 1-3 malignant LNs, and N2 for 4 or more [4, 9]. This study is unique compared to previous work as it combines patients both with and without pre-operative CRT into the same cohort.

**Fig. 1:**
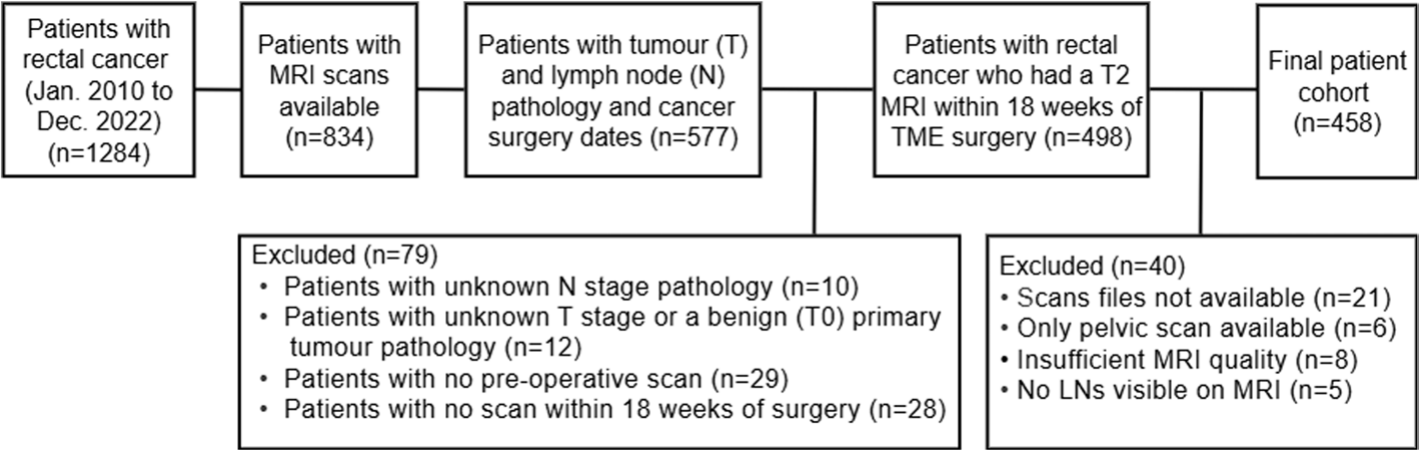
Flow diagram of enrolment of patients in the research dataset

The dataset excluded 19 patient scans due to insufficient MRI quality (n=8), lack of a small field of view axial or coronal scan (n=6), and when there were not any LNs visible on axial or coronal (n=5). In practice, radiologists would use both axial and coronal field of view scans, and so 28 coronal scans were included in cases where they were more appropriate. Reasons include: when there was not an axial scan (n=5), when there were not any LNs visible on the axial but there was on the coronal (n=5), and lastly, some N1+ axial scans were swapped for coronal when there were more visible suspicious LNs on the coronal (n=18). Only one scan was used per patient to avoid bias from repeated LNs. A full breakdown of the inclusion and exclusion is provided in Fig. 1. The clinical and pathological summary of the dataset is given in Table 1.

**Table 1:** Clinical characteristics of the patient cohorts with percentages in brackets for: the full dataset (All), patients receiving radiotherapy or chemoradiotherapy (CRT) before surgery, patients that did not have any pre-operative treatment (non-CRT), and the clinical validation study cohort (Validation)

Characteristic	All (n=458)	non-CRT (n=314)	CRT (n=144)	Validation (n=50)
Age (Mean ± SD)	68.3 ± 11.3	68.6 ± 11.4	67.6 ± 11.2	67.1 ± 10.7
Sex				
Male	306 (66.8)	210 (66.9)	96 (66.7)	37 (74)
Female	152 (33.2)	104 (33.1)	48 (33.3)	13 (26)
Number of Comorbidities	2.3 ± 1.2	2.3 ± 1.2	2.3 ± 1.2	2.1 ± 1.0
Tumour Pathology				
T1	48 (10.5)	37 (11.8)	11 (7.6)	2 (4)
T2	146 (31.9)	121 (38.5)	25 (17.4)	19 (38)
T3	199 (43.4)	115 (36.6)	84 (58.3)	20 (40)
T4	65 (14.2)	41 (13.1)	24 (16.7)	9 (18)
Node Pathology				
N0	279 (60.9)	212 (67.5)	67 (46.5)	29 (58)
N1	129 (28.2)	70 (22.3)	59 (41)	16 (32)
N2	50 (10.9)	32 (10.2)	18 (12.5)	5 (10)
Metastasis Pathology				
M0	346 (75.5)	238 (75.8)	108 (75)	39 (78)
M1	33 (7.2)	20 (6.4)	13 (9)	4 (8)
MX (unknown)	79 (17.2)	56 (17.8)	23 (16)	7 (14)
Number of LNs on MRI				
(N (Mean ± SD))	3760 (8.2 ± 4.9)	2639 (8.4 ± 4.9)	1121 (7.8 ± 4.9)	428 (8.6 ± 4.4)
Short-axis Diameter (*mm*)	5.53 ± 2.35	5.58 ± 2.26	5.43 ± 2.54	4.40 ± 2.54
Long-axis Diameter (*mm*)	7.60 ± 3.08	7.65 ± 2.98	7.46 ± 3.30	9.75 ± 3.00
Short / Long-axis Ratio	0.72 ± 0.08	0.73 ± 0.08	0.72 ± 0.09	0.73 ± 0.07

### MRI preprocessing and augmentation

Preprocessing of the MRI scans was performed using TorchIO [10] to crop and normalise lymph node patches with shape ($32 \times 32 \times 4$) voxels. The pipeline included histogram intensity scaling, clipping values outside of the [1, 99] percentiles, and resampling intensity values to [0,1] with a consistent scan orientation and a standard voxel size of ($0.573mm \times 0.573mm \times 3.3mm$). 3D MRI patches correspond to an anatomical region of interest with a volume of $4438.2mm^3$. The largest diameter LN patches were selected for each patient, with a limit of 15, and empty spaces were filled with zero padding. The model input shape was ($\text {batch size} \times 15 \times 32 \times 32 \times 4$).

Data augmentation was used to increase dataset variability and improve the robustness and generalisability of the feature encoder models. Spatial augmentations included horizontal, vertical and depth-wise flips, as well as translations to shift the patch centre by up to 6 voxels, and rotations up to 10 degrees to capture different context from the surroundings. Intensity-based augmentations applied included: random Gaussian noise, random blur, gamma correction, and bias field distortion to simulate magnetic field inhomogeneities.

### Method

**Fig. 2:**
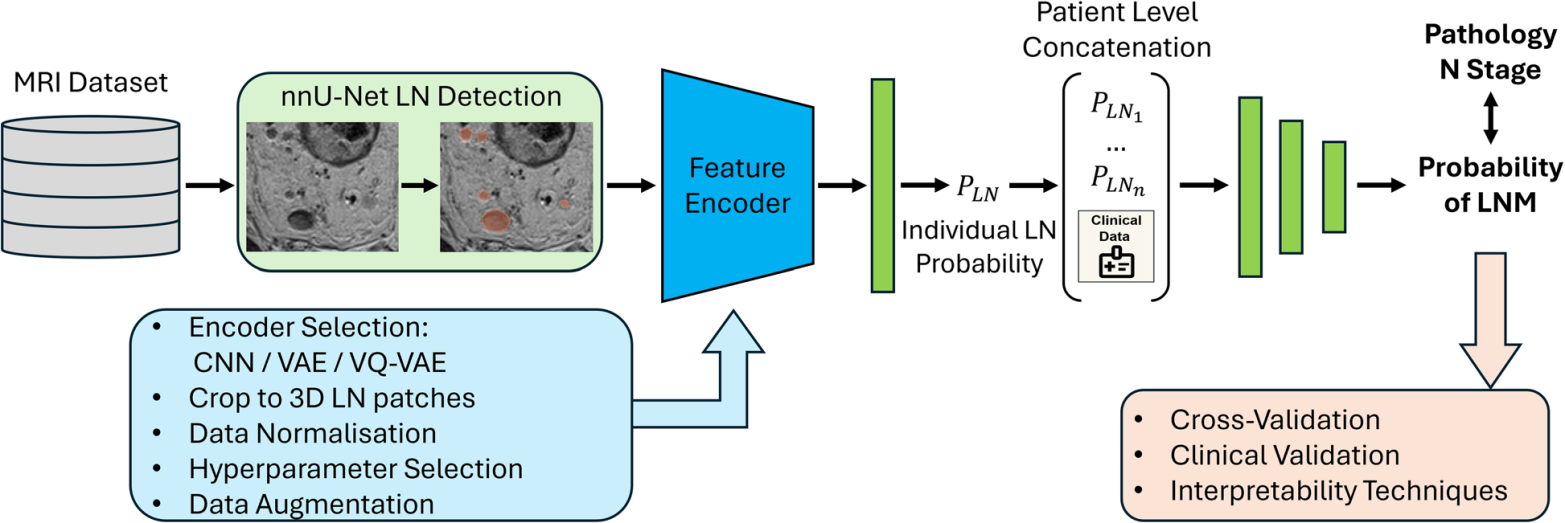
Workflow and architecture diagram of radiological LNM staging model

The end-to-end LNM staging model is visualised in Fig. 2. The key component investigated was the feature encoder backbone, with optimal MLP hyperparameters for each encoder. The overall pipeline was standardised, including preprocessing, segmentation, augmentation, and multiple instance learning with clinical data.

#### Automatic LN detection

The out-of-the-box segmentation tool nnU-Net [5] was applied to detect lymph nodes across the 458 pre-operative MRI scans. The framework applies preprocessing and configures U-Net models using an architecture and optimised hyperparameters based on experiments across a variety of medical datasets, with many resulting in state-of-the-art performance [11].

The LNs annotations were completed manually by a postgraduate student using the open-source software 3D Slicer [12, 13]. Initially, a random set of 200 scans were labelled and used to train and test the nnU-Net model. To ensure annotation quality, a sample of 34 scans (17%) was validated by a consultant gastrointestinal (GI) radiologist. Following the review, the total number of LNs in the sample changed from 303 to 271 with 46 removed and 14 added, corresponding to a 94.8% sensitivity at detecting LNs. Additionally, 17 scans (50%) were correctly labelled and unchanged.

The completed annotations were used as training data for a 3D nnU-Net automatic lymph node segmentation model. An iterative approach was used to firstly check and update a random sample of the segmentation files generated by nnU-Net on unlabelled scans before retraining the nnU-Net with an expanded training dataset. The increased cohort size yielded a higher Dice score each time, improving the efficiency of the labelling process.

#### 3D feature encoder models

This study investigated and compared feature three 3D feature encoder models including: (1) Convolutional Neural Network (CNN), (2) Variational Autoencoder (VAE), and (3) Vector Quantised VAE (VQ-VAE). These models were used to generate feature representations for each lymph node, which were then used in combination with clinical and radiomic features in a multiple instance learning approach using multi-layer perceptrons to produce patient-level lymph node metastasis predictions.

Convolutional Neural Networks (CNN) [14] are an established foundational architecture, well known for their strong performance in medical image classification. Models extract hierarchical spatial features from image data, and they can be particularly effective when working with limited data, especially if combined with transfer learning techniques [15].

Variational Autoencoders (VAE) [16] are a generative encoder-decoder architecture which encodes input data as a latent vector representation and then uses this to reconstruct the input. The self-supervised learning approach trains a CNN-based encoder to learn general features useful for reconstruction, and the latent vectors can be used for classification purposes. VAEs have demonstrated performance comparable to state-of-the-art methods in several medical imaging studies, including pre-operative detection of breast cancer [17] and lung cancer [18] on public datasets. The VAE learns a feature representation for each MRI lymph node patch and then reconstructs the image using a reverse architecture. The latent vector size of the final VAE model was 432 numbers, acting as a set of coordinates in a structured latent space that allows for interpretable insights.

Lastly, Vector Quantised VAEs (VQ-VAE) [19] are a discrete variant of the standard VAE, which split input data into a grid of patches, and then learns to match each patch to a discrete latent vector, or ‘codebook feature’, from a learned codebook. Analogously, it is like matching jigsaw pieces to reconstruct the input. These latent vectors may be interpreted to show what they are encoding, and what the model recognises as the important general features of the data, providing insights into the underlying structure. VQ-VAEs match the codebook features to specific parts of the MRI lymph node images, allowing spatial insights into which features are important in classification. The classification model uses the index of the latent vectors only, and there are 64 numbers per lymph node representation in an $8 \times 8$ grid.

The final VAE and VQ-VAE MRI reconstructions were evaluated qualitatively and quantitatively with the average Structural Similarity Index Measure (SSIM) [20], Mean Squared Error (MSE), Mean Absolute Error (MAE), Peak Signal-to-Noise Ratio (PSNR), and Learned Perceptual Image Patch Similarity (LPIPS) [21].

#### Lymph node metastasis classification

The classification multiple instance learning (MIL) multi-layer perceptron (MLP) model is visualised in Fig. 2. The MLP layers include 1D Batch Normalisation, dropout and GELU activation [22]. The final layer of each MLP was a fully connected layer followed by a sigmoid activation function. A patch MLP was used to refine the feature representations along with treatment data, and size, border and texture characteristics into individual LN predictions. The patient-level representation included up to 15 LN predictions and the full set of clinical features, this was then input to a patient MLP for the final binary N-stage diagnosis.

#### Clinical features

Patient clinical data included age, sex (binary male or female), the primary tumour stage (T1-T4), treatment type given before the scan (binary indicators for chemotherapy and radiotherapy), and comorbidities. Two comorbidities were included due to their established association with rectal cancer: malabsorption or inflammatory bowel disease [23], and obesity [24].

LN shape and texture morphology radiomics features were included based on the clinically relevant staging criteria used by radiologists and included in TNM staging guidelines [4, 9]. Size and border irregularity features were extracted from the LN segmentation masks, and texture features used the MRI signal intensity values from within the LN masks. Firstly, size features included the long and short-axis diameter and their ratio. Border irregularity metrics included the Dice score between the mask and its convex hull (Dice), where a lower Dice score indicates a more irregular border, and the ratio of low energy to total energy from a 3D Fast Fourier Transform (FFT). The low frequencies correspond to a smooth border and higher frequencies show irregularities in the LN border, so a higher proportion of low frequencies (higher FFT) means there is a more regular border and a lower FFT indicates a more irregular border. Texture features included the coefficient of variation (CV), which is the standard deviation divided by the mean, and Shannon entropy (Entropy). Both measure variation in the signal intensity within the LNs, and a higher score corresponds to more variation. These features aim to detect a heterogeneous signal intensity pattern, which is a known malignant characteristic [9].

Lastly, a feature was included to capture the chemical shift artifact, caused by fat-water interfaces in benign lymph nodes. This artifact appears on MRI along the lymph node border, with a lighter border on one side and darker on the other. This happens due to the fat content of benign lymph nodes, whereas malignant lymph nodes generally do not contain fat and therefore will not have a chemical shift artifact. This criterion has been shown to be effective in predicting lymph node status on rectal cancer MRI [25], however, it is not included in the current TNM staging guidelines [9]. Chemical shift was measured using the range of intensity values along the LN border.

These clinical features were normalised to the range [0, 1] using the min and max across the training dataset. The LN features and treatment variables were concatenated with the MRI LN patch feature representations before the patch-level MLP. Then patient-level clinical variables were combined with individual LN probabilities and input to the patient-level classification model. The lymph node radiomics features were summarised at the patient level and also concatenated with the patient representation. A hyperparameter was used to determine how to summarise the LN shape and texture morphology features, either using the mean, max, maximum LN short-axis diameter or maximum probability LN.

#### Ablation

The key model components of the proposed model were evaluated using cross-validation and a strategy that tests the importance of the clinical and deep learning features. First, the results of the three feature encoder models from the first stage of training were reported. These models used only the LN radiomic features and deep learning representations, excluding the patient-level clinical data. Next, two simplified models were trained to demonstrate the benefit of incorporating deep learning features extracted from the MRI data. The Patient Logistic Regression model used only the patient-level clinical data, and the Patient+LN MLP used both lymph node radiomic features and patient-level clinical data. The Patient+LN model pipeline learns a feature representation for the patient clinical data and for each lymph node using separate MLPs and then combines them in a final MLP that gives probabilities for each lymph node. The final binary N-stage prediction was then taken as the maximum probability from the set of lymph nodes.

#### Implementation details

The code for the CNN, VAE and MLP models was bespoke and investigated thoroughly to maximise performance. The VAE model uses convolutional upsampling instead of convolutional transpose, and a custom loss function that includes the SSIM loss with trained hyperparameter weightings. The VQ-VAE model was adapted from the MONAI implementation [26]. Model experiments were completed using an NVIDIA A5000 24 GB GPU, with results recorded and compared using Weights and Bias (WandB) [27]. To reduce the computational complexity, feature encoder models were trained using half precision (16-bit floating point) and MLP classifiers using single precision (32-bit). The model required 51 seconds per case to perform preprocessing, segmentation and classification of a single MRI scan on a GPU, and no effort was made to optimise the code.

The VAE and VQ-VAE models were trained in a self-supervised manner, and the model with the highest average test SSIM between the original images and the reconstructions was selected from around 50 candidates each. The MIL MLP classification model for the three feature encoder models was then trained using the lymph node pathology and Focal Loss [28] to help focus the model on the positive class and difficult cases. The pre-trained VAE models were fixed, whereas the CNN was trained end-to-end with the MLP. MLP models were trained in two stages, first using the MRI patches, LN features and treatment data, with the individual LN probabilities saved for the highest test balanced accuracy model. The final stage used the saved LN patch probabilities, which had a small amount of Gaussian noise added on each epoch for the training data, and included all clinical data. This strategy was used so that the model first learns informative LN probabilities and does not overfit to the clinical data as it did in initial experiments.

Model agnostic hyperparameters included the learning rate, weight decay, batch size, dropout probability (0.1 to 0.2), focal alpha (0.6-0.75 to focus on the positive class), gradient accumulation steps (to average over 1-3 batches), the number of synthetic patients created using augmentation, and the number of layers and the hidden layer size in the patch and patient MLPs.

A large Bayesian search was used to select the best hyperparameters for each model and stage. The patient MLP was fixed to 2 layers with a hidden size of 16 during the first stage of training to ensure that the model focused on finding the best patch MLP. Five-fold cross-validation was then completed using the multiple stage approach on each fold separately. Important selected hyperparameters used in cross-validation for the patch and patient MLPs are displayed in Table 2.

**Table 2:** Important hyperparameters for the patch and patient MLP models including: the learning rates (patch and patient learning rates (lrs)), batch size (patch and patient batch sizes), the number of synthetic patients (num synth) created using augmentation and used in the patch and patient MLP models, and the number of layers and hidden layer sizes for both models

Model	lrs	Batch sizes	Num synth	Patch layers	Patch dim	Patient layers	Patient dim
VAE	$7\cdot 10^{-4}$ & $2\cdot 10^{-3}$	64 & 32	8 & 10	2	256	4	48
VQ-VAE	$1.5\cdot 10^{-3}$ & $7\cdot 10^{-4}$	48 & 48	10 & 5	3	256	4	64
CNN	$1.5\cdot 10^{-3}$ & $2\cdot 10^{-3}$	16 & 64	8 & 5	3	512	4	32

### Clinical validation study

A clinical validation study was completed by three radiologists independently at LTHT, a large tertiary oncology unit in England. Firstly, two radiologists with 5 and 6 years of experience, and a consultant radiologist with 4 years of experience as a consultant in a colorectal cancer multidisciplinary team (MDT). The study involved the radiologists reviewing scans and submitting an initial staging before viewing the AI model predictions, then viewing the AI output and submitting a final N staging. Direct comparisons were made between the model and expert radiological staging across a random sample of 50 scans from the test set of the best individual model (VQ-VAE). The sample was broadly representative of the dataset as shown in Table 1, with 36 (72%) non-CRT patients compared to 68.5% across the dataset. The radiologists viewed the axial, coronal and sagittal fields of view and completed an N staging assessment in line with standard clinical practice. A final staging was then reported after viewing the model binary N staging prediction and individual LN patch scores alongside the corresponding LN MRI images.

The method of comparison included reporting the summary performance metrics (sensitivity, specificity, accuracy, F1 and balanced accuracy), summarising agreement and disagreement between radiologists and the model, reporting the confusion matrix of each radiologist and the majority vote, and lastly using the exact McNemar test and a generalised linear mixed model (GLMM) analysis to determine whether the model had a statistically significant improvement in classification compared to the radiologists. The McNemar test evaluates paired outcomes (radiologist and AI results), testing the null hypothesis that the marginal proportions are equal, with an alternative hypothesis that the true odds ratio is not equal to 1, which is equivalent to finding if there is a statistically significant difference in performance. The GLMM with a binomial distribution and logit link function was fitted with random intercepts for the radiologist reader and scan to account for the variability in radiologist skill and scan difficulty, and a fixed effect for whether the prediction was from a radiologist or the AI model as follows,

$$\begin{aligned} \begin{array}{c} \text {correct}_{ij} = \beta _0 + \beta _1 \,\text {AI}_{ij} + u_i + v_j \\ \text {where } u_i \sim \mathcal {N}(0, \sigma ^2_{\text {radiologist}}), \quad v_j \sim \mathcal {N}(0, \sigma ^2_{\text {scan}}) \end{array} \end{aligned}$$

The dataset consisted of 300 observations of reader-scan (*i*,*j*) pairs with binary variables for whether the AI prediction was used or not and whether the prediction was correct. This included 150 observations for the 50 scans with three radiologist predictions, with $\text {AI}_{ij}=0$, then a further dataset of 150 using AI, with $\text {AI}_{ij}=1$.

This clinical validation study was completed to see how the model compares to clinical practice and whether the radiological staging model has the potential to improve radiological staging when compared to current processes.

### Interpretability

Interpretability experiments were conducted using the trained VAE and VQ-VAE latent vectors and the corresponding LN radiomic features to interpret the underlying structure of the models. Using the individual lymph node probabilities from the best model, the dataset was split into suspicious and benign subsets with high ($p>0.6$) and low ($p<0.4$) probability, excluding any high probability LNs with N0 pathology.

Firstly, VQ-VAE codebook features were investigated to identify codes that were significantly associated with the suspicious or benign groups. Hypothesis tests were performed using Fisher’s exact test with a Benjamini-Hochberg false discovery rate (FDR) correction of *p*-values, to adjust for the increased likelihood of false positives when many tests are performed. Comparisons for each code were made between the high probability group versus all LNs, low versus all, and high versus low. Significant codebook features were visualised in 2D using dimensionality reduction techniques: Principal Component Analysis (PCA) and t-distributed Stochastic Neighbour Embedding (t-SNE) [29]. Odds ratios were calculated to show the increased/decreased likelihood that a given codebook feature appears in the set of high-probability LNs compared to the full dataset. Lastly, statistics were generated to show what specific codes are associated with, including lymph node size, border and texture.

VAE interpretability experiments included latent traversals along the average direction between the centroid of low probability LNs ($p\le 0.33$ and negative pathology) and high probability LNs ($p\ge 0.66$ and positive pathology) in the VAE latent space. The average latent vector position was found for each group, and then the difference was taken to find the direction vector from low to high probability. Step sizes may be chosen through trial and error, in this case, a step size of 0.5 of the direction vector was applied for the examples displayed. The direction was iteratively applied to the latent vector of an MRI patch, with each updated latent vector decoded by the VAE and visually displayed for each step to show that the model has disentangled malignant characteristics.

## Results

### nnU-Net segmentation

**Fig. 3:**
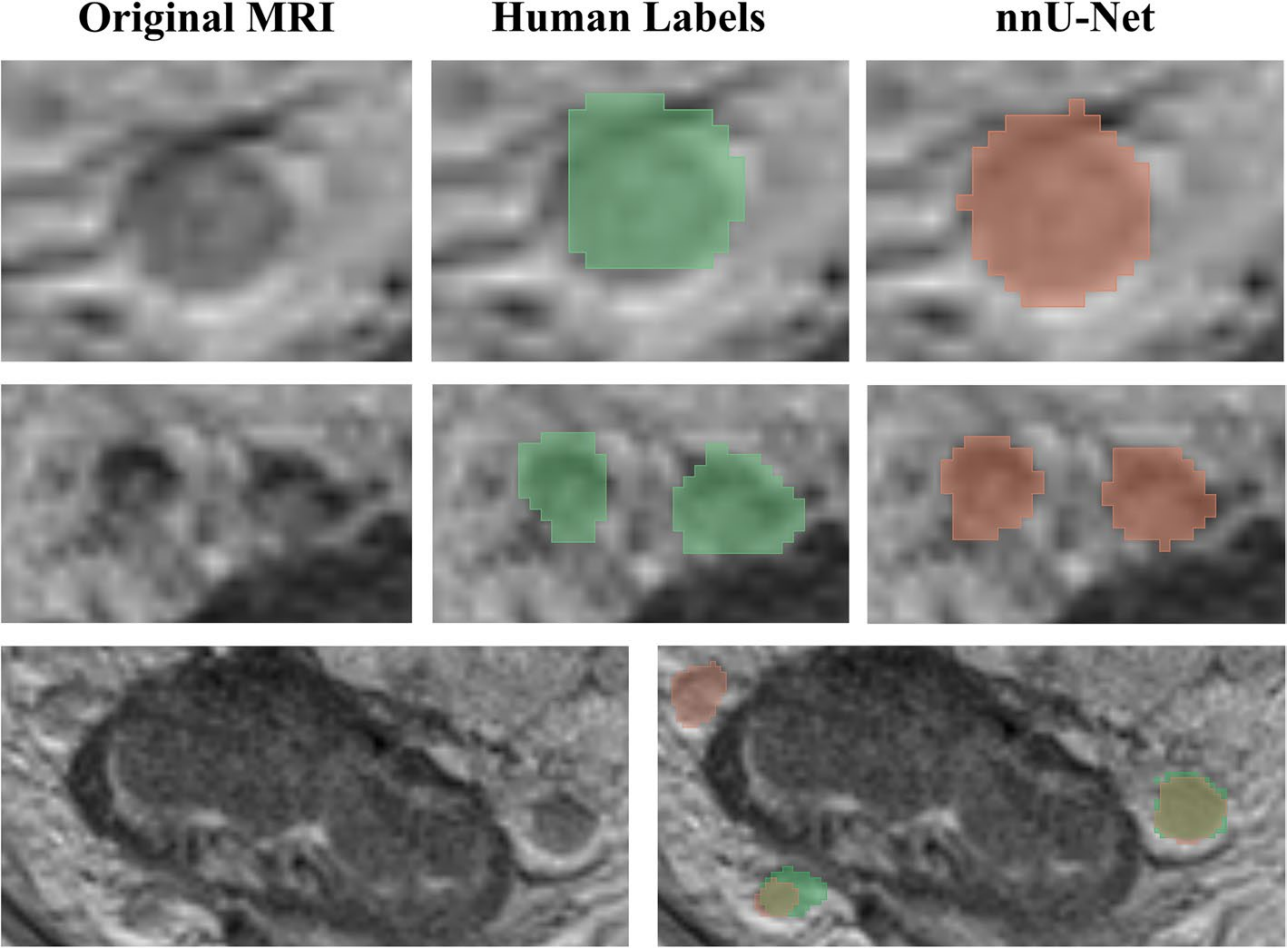
Example segmentation results comparing the human annotator with the AI segmentation algorithm nnU-Net [5]

The final nnU-Net model achieved a Dice score of 0.73 on the LN annotations and 0.999 Dice score on the background. From the 458 MRI scans, 160 (34.9%) of the nnU-Net predicted masks were left unchanged, and on the remaining scans, the annotator’s cumulative updates included 134 LNs removed and 501 added or updated, corresponding to 16.9% of the 3760 LNs identified in total. A comparison of the nnU-Net segmentations with the human annotator is shown in Fig. 3. The model was very capable at detecting obvious LNs, and often captured the LN border more precisely than the annotator. These results suggest that the task of detecting mesorectal lymph nodes on MRI is manageable and straightforward to implement with nnU-Net.

### VAE and VQ-VAE MRI reconstructions

**Fig. 4:**
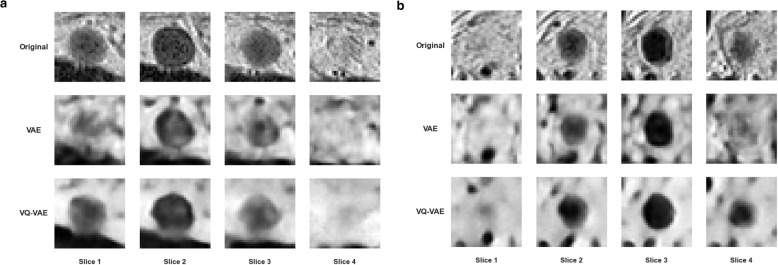
VAE and VQ-VAE reconstructions of MRI lymph node patches

Table 3 reports the test set reconstruction metrics from the VAE and VQ-VAE models, averaged across five cross-validation folds, and Fig. 4 shows example test set reconstructions by both models. These results show that the two models accurately capture the lymph node size, shape and signal intensity patterns.

**Table 3:** Reconstruction metrics for VAE and VQ-VAE models, with arrows indicating if higher ($\uparrow$) or lower ($\downarrow$) values correspond with better quality reconstructions. Bold values indicate the best performance for each metric

Model	SSIM $\boldsymbol{\uparrow }$	MSE $\boldsymbol{\downarrow }$	MAE $\boldsymbol{\downarrow }$	PSNR $\boldsymbol{\uparrow }$	LPIPS $\boldsymbol{\downarrow }$
VAE	$\boldsymbol{0.5685 \pm 0.0051}$	$0.0136 \pm 0.0007$	$0.0866 \pm 0.0024$	$18.7715 \pm 0.2490$	$\boldsymbol{0.0763 \pm 0.0029}$
VQ-VAE	$0.4900 \pm 0.0071$	$\boldsymbol{0.0101 \pm 0.0003}$	$\boldsymbol{0.0740 \pm 0.0012}$	$\boldsymbol{20.0459 \pm 0.1507}$	$0.1052 \pm 0.0052$

**Fig. 5:**
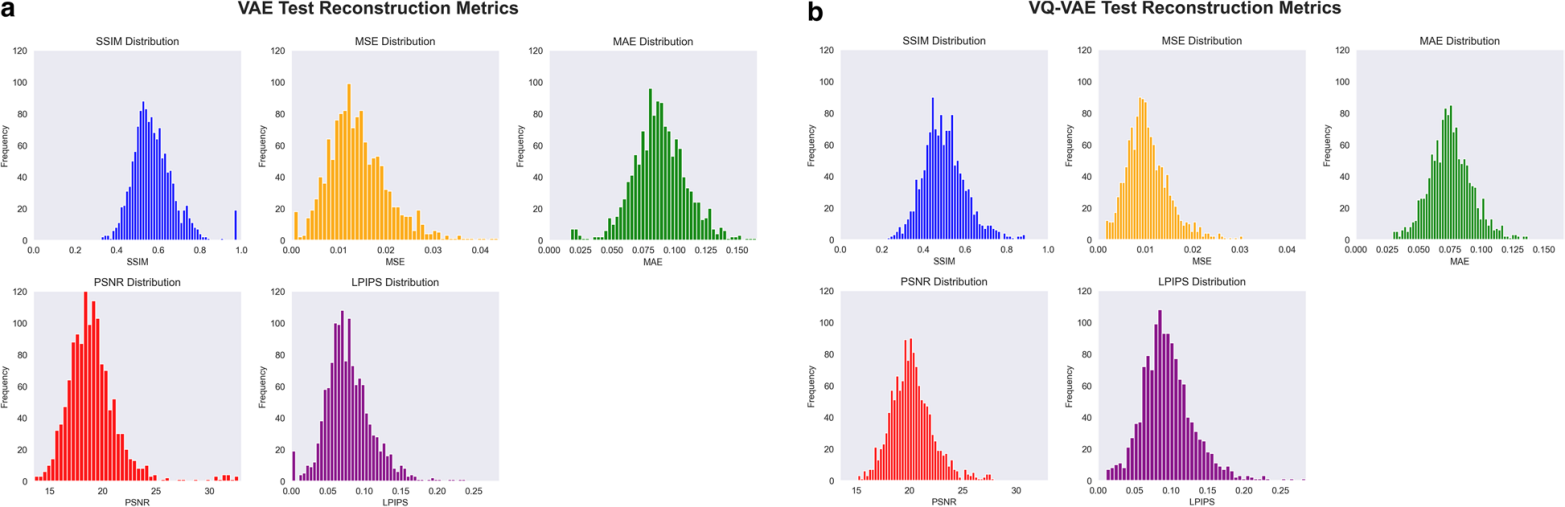
Distribution of test set reconstruction quality metrics (SSIM, MSE, MAE, PSNR, and LPIPS) for the VAE and VQ-VAE models

The histograms of test set reconstruction metrics are shown in Fig. 5. These graphs show approximately Gaussian distributions, highlighting differences in reconstruction performance across the dataset.

### Evaluation of LNM model

#### Classification results

The most clinically important metric is sensitivity, as it represents how many LNM cases were identified according to the pathology, and patients with suspicious LNs detected on MRI are generally considered for neo-adjuvant CRT treatment [6]. In this context, a lower sensitivity would correspond to more patients with undetected LNM, potentially leading to inappropriate treatment and worse patient outcomes. Models were selected based on balanced accuracy (BA), the average of sensitivity and specificity, with an emphasis on achieving high sensitivity as the model is intended to be a screening tool where identifying positive cases is the most important.

Cross-validated results are reported in Table 4, with a breakdown of the performance metrics within the two treatment groups: patients receiving neo-adjuvant CRT treatment, and patients not receiving neo-adjuvant treatment (non-CRT).

**Table 4:** Lymph node metastasis classification cross-validation performance metrics, split by patient cohorts with and without neo-adjuvant radiotherapy or chemoradiotherapy (CRT) treatment

Group	Model	AUC	Sensitivity	Specificity	Accuracy	F1	BA
Combined	VAE	$0.8116 \pm 0.019$	$\mathbf {0.8666} \boldsymbol{\pm } \mathbf {0.052}$	$0.6919 \pm 0.047$	$0.7598 \pm 0.023$	$0.7382 \pm 0.022$	$0.7793 \pm 0.023$
	VQ-VAE	$\mathbf {0.8282} \boldsymbol{\pm } \mathbf {0.042}$	$0.8660 \pm 0.051$	$\mathbf {0.7240} \boldsymbol{\pm } \mathbf {0.037}$	$\mathbf {0.7795} \boldsymbol{\pm } \mathbf {0.031}$	$\mathbf {0.7541} \boldsymbol{\pm } \mathbf {0.036}$	$\mathbf {0.7950} \boldsymbol{\pm } \mathbf {0.033}$
	CNN	$0.8102 \pm 0.058$	$0.8546 \pm 0.037$	$0.7098 \pm 0.027$	$0.7664 \pm 0.027$	$0.7407 \pm 0.031$	$0.7822 \pm 0.028$
CRT	VAE	$0.7828 \pm 0.068$	$\mathbf {0.9608} \boldsymbol{\pm } \mathbf {0.059}$	$0.3868 \pm 0.156$	$0.6949 \pm 0.063$	$0.7722 \pm 0.037$	$0.6738 \pm 0.073$
	VQ-VAE	$0.8003 \pm 0.061$	$0.9483 \pm 0.029$	$0.4956 \pm 0.168$	$0.7370 \pm 0.077$	$0.7964 \pm 0.050$	$0.7220 \pm 0.082$
	CNN	$\mathbf {0.8177} \boldsymbol{\pm } \mathbf {0.067}$	$0.9208 \pm 0.110$	$\mathbf {0.6297} \boldsymbol{\pm } \mathbf {0.105}$	$\mathbf {0.7846} \boldsymbol{\pm } \mathbf {0.053}$	$\mathbf {0.8189} \boldsymbol{\pm } \mathbf {0.053}$	$\mathbf {0.7752} \boldsymbol{\pm } \mathbf {0.051}$
non-CRT	VAE	$0.8067 \pm 0.038$	$0.7952 \pm 0.068$	$0.7880 \pm 0.066$	$0.7898 \pm 0.031$	$0.7114 \pm 0.029$	$0.7916 \pm 0.024$
	VQ-VAE	$\mathbf {0.8272} \boldsymbol{\pm } \mathbf {0.071}$	$\mathbf {0.8038} \boldsymbol{\pm } \mathbf {0.106}$	$\mathbf {0.7977} \boldsymbol{\pm } \mathbf {0.083}$	$\mathbf {0.7997} \boldsymbol{\pm } \mathbf {0.053}$	$\mathbf {0.7234} \boldsymbol{\pm } \mathbf {0.071}$	$\mathbf {0.8007} \boldsymbol{\pm } \mathbf {0.053}$
	CNN	$0.7868 \pm 0.075$	$0.8028 \pm 0.073$	$0.7360 \pm 0.029$	$0.7580 \pm 0.026$	$0.6821 \pm 0.043$	$0.7694 \pm 0.035$
	Radiologists [7]	0.7894	$0.7300 \pm 0.030$	$0.7500 \pm 0.020$			0.7400
SOTA (non-CRT)	Xia et al. [30]	$0.8100 \pm 0.045$	$0.7020 \pm 0.072$	0.8000			0.7510
	Wan et al. [31]	$0.7900 \pm 0.100$	1.000	$0.6600 \pm 0.110$	$0.7300 \pm 0.090$		0.8300
	Yang et al. [32]	$0.7520 \pm 0.122$	0.7830	0.6740	0.7120		0.7285
	Zhao et al. [33]	$0.7820 \pm 0.145$	$0.7140 \pm 0.197$	$0.8080 \pm 0.151$	$0.7660 \pm 0.120$		0.7610

Results include cross-validation scores, comparisons with state-of-the-art (SOTA) performance of deep learning rectal cancer lymph node metastasis MRI evaluation on external MRI datasets, and the average performance of radiologists on MRI calculated in a systematic review [7]. Bold values indicate the best performance for each metric in each group

**Fig. 6:**
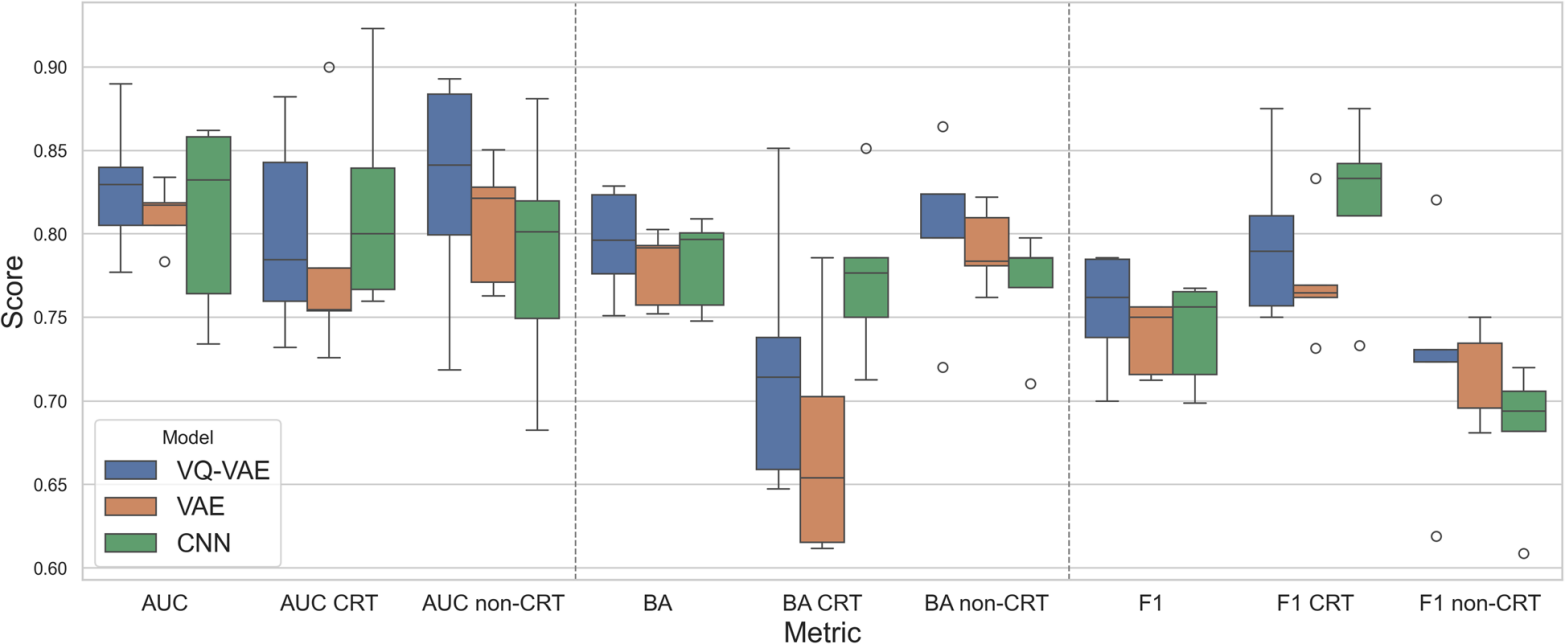
Cross-validation results for all models with distribution across metrics AUC, balanced accuracy (BA) and F1 score for all patients and a breakdown for the chemoradiotherapy (CRT) and non-CRT patient groups

Figure 6 displays boxplots for the three cross-validated models with a breakdown over three metrics (AUC, balanced accuracy, and F1 score) and across the three key patient groups defined in Table 1. The boxplot is set up with a box covering the lower quartile (25%), median (50%), upper quartile (75%), and whiskers with 1.5 interquartile range (IQR) above the upper quartile and below the lower quartile, with outliers represented as circles. The plots show that the VQ-VAE, VAE, and CNN models had relatively consistent performance, with mostly similar boxplot sizes across the three models for the same metric and patient group.

#### Ablation

Table 5 displays the results of the ablation study. The first section shows the cross-validated results of the main models without using patient-level clinical data. These results compare directly with the final model classification results displayed in Table 4 and demonstrate that the clinical data significantly improves the balanced accuracy for all models. The next component of the ablation study includes two simplified models which demonstrate the baseline performance without deep learning features and only using clinical and radiomics data. The Patient+LN MLP model performance is much better than the Patient Logistic Regression model only using patient-level clinical data for almost all metrics, suggesting that the radiomic features are discriminative and important to the classification. However, sensitivity is the most important metric and the Patient+LN model had a much lower sensitivity of 67.5% compared to 86.6% for the VQ-VAE, suggesting that the deep learning features are important for detecting malignancy. Compared to Table 4, the best model (VQ-VAE) had 2.9% higher balanced accuracy than the Patient+LN MLP, and 7.2% higher for the non-CRT group. Overall, this ablation study demonstrates that the deep learning features are complementary to the clinical and radiomics features, and that deep learning methods can achieve the higher sensitivity essential for improving patient outcomes.

**Table 5:** Ablation study showing the classification results of the model without patient-level clinical data, and results for models not using deep learning and only the patient clinical data (Patient) with or without lymph node radiomic features (LN)

Group	Model	AUC	Sensitivity	Specificity	Accuracy	F1	BA
No Clinical Data	VAE	$0.7795 \pm 0.057$	$\mathbf {0.8556} \boldsymbol{\pm } \mathbf {0.068}$	$0.6382\pm 0.093$	$0.7226 \pm 0.043$	$\mathbf {0.7076} \boldsymbol{\pm } \mathbf {0.030}$	$\mathbf {0.7469} \boldsymbol{\pm } \mathbf {0.033}$
	VQ-VAE	$\mathbf {0.7962} \boldsymbol{\pm } \mathbf {0.055}$	$0.7879 \pm 0.084$	$\mathbf {0.6812} \boldsymbol{\pm } \mathbf {0.079}$	$\mathbf {0.7227} \boldsymbol{\pm } \mathbf {0.062}$h	$0.6896 \pm 0.069$	$0.7345 \pm 0.062$
	CNN	$0.7344 \pm 0.011$	$0.7371 \pm 0.045$	$0.6767\pm 0.091$	$0.7008 \pm 0.038$	$0.6590 \pm 0.014$	$0.7069 \pm 0.024$
Combined	Patient Logistic Regression	$0.7240\pm 0.041$	$0.4752\pm 0.055$	$0.8285\pm 0.062$	$0.690\pm 0.042$	$0.5450\pm 0.056$	$0.6519\pm 0.042$
	Patient+LN MLP	$\mathbf {0.8339} \boldsymbol{\pm } \mathbf {0.045}$	$\mathbf {0.6745} \boldsymbol{\pm } \mathbf {0.082}$	$\mathbf {0.8566} \boldsymbol{\pm } \mathbf {0.046}$	$\mathbf {0.7861} \boldsymbol{\pm } \mathbf {0.039}$	$\mathbf {0.7097} \boldsymbol{\pm } \mathbf {0.062}$	$\mathbf {0.7656} \boldsymbol{\pm } \mathbf {0.046}$
CRT	Patient Logistic Regression	$0.5825\pm 0.120$	$0.7700\pm 0.162$	$0.4505\pm 0.208$	$0.6188\pm 0.084$	$0.6796\pm 0.072$	$0.6103\pm 0.082$
	Patient+LN MLP	$\mathbf {0.8549} \boldsymbol{\pm } \mathbf {0.075}$	$\mathbf {0.8175} \boldsymbol{\pm } \mathbf {0.057}$	$\mathbf {0.7473} \boldsymbol{\pm } \mathbf {0.147}$	$\mathbf {0.7854} \boldsymbol{\pm } \mathbf {0.065}$	$\mathbf {0.8043} \boldsymbol{\pm } \mathbf {0.057}$	$\mathbf {0.7824} \boldsymbol{\pm } \mathbf {0.070}$
non-CRT	Patient Logistic Regression	$0.7338\pm 0.044$	$0.2538\pm 0.098$	$\mathbf {0.9483} \boldsymbol{\pm } \mathbf {0.019}$	$0.7231\pm 0.040$	$0.3684\pm 0.129$	$0.6010\pm 0.058$
	Patient+LN MLP	$\mathbf {0.8080} \boldsymbol{\pm } \mathbf {0.058}$	$\mathbf {0.5667} \boldsymbol{\pm } \mathbf {0.104}$	$0.8915\pm 0.027$	$\mathbf {0.7865} \boldsymbol{\pm } \mathbf {0.044}$	$\mathbf {0.6296} \boldsymbol{\pm } \mathbf {0.092}$	$\mathbf {0.7291} \boldsymbol{\pm } \mathbf {0.060}$

Bold values indicate the best performance for each metric in each group

#### Individual model validation

Confusion matrices for the best individual VQ-VAE, VAE and CNN models are displayed in Table 6, and individual model performance results are displayed in Table 7. These models were the best from individual training runs, with a test set size of 138. The hyperparameters from these models (see Table 2) were used in the cross-validation runs. The results of the individual models indicate that the VQ-VAE has the best overall performance according to balanced accuracy and AUC, followed by the VAE and CNN, which is consistent with the cross-validation results in Table 4.

**Table 6:** Confusion matrices for VQ-VAE, VAE, and CNN models

	VQ-VAE		VAE		CNN	
	Predicted negative	Predicted positive	Predicted negative	Predicted positive	Predicted negative	Predicted positive
Actual Negative	64	20	62	22	64	20
Actual Positive	5	49	5	49	7	47

**Table 7:** Lymph node metastasis classification individual model performance metrics, split by patient cohorts with and without neo-adjuvant radiotherapy or chemoradiotherapy (CRT) treatment

Group	Model	AUC	Sensitivity	Specificity	Accuracy	F1	BA
Combined	VAE	0.8538	**0.9074**	0.7381	0.8043	0.7840	0.8228
	VQ-VAE	**0.8829**	**0.9074**	**0.7619**	**0.8188**	**0.7967**	**0.8347**
	CNN	0.8719	0.8704	**0.7619**	0.8043	0.7769	0.8161
CRT	VAE	**0.9167**	**1.000**	0.6250	0.8378	0.8750	0.8125
	VQ-VAE	0.8839	**1.000**	0.5625	0.8108	0.8571	0.7813
	CNN	0.9077	**1.000**	**0.7500**	**0.8919**	**0.9130**	**0.8750**
non-CRT	VAE	0.8275	**0.8485**	0.7647	0.7921	0.7273	0.8066
	VQ-VAE	**0.8676**	**0.8485**	**0.8088**	**0.8218**	**0.7568**	**0.8287**
	CNN	0.8373	0.7879	0.7647	0.7723	0.6933	0.7763
	Radiologists [7]	0.7894	$0.73 \pm 0.03$	$0.75 \pm 0.02$			0.7400
SOTA (non-CRT)	Xia et al. [30]	$0.8100 \pm 0.045$	$0.7020 \pm 0.072$	0.8000			0.7510
	Wan et al. [31]	$0.7900 \pm 0.100$	1.000	$0.6600 \pm 0.110$	$0.7300 \pm 0.090$		0.8300
	Yang et al. [32]	$0.7520 \pm 0.122$	0.7830	0.6740	0.7120		0.7285
	Zhao et al. [33]	$0.7820 \pm 0.145$	$0.7140 \pm 0.197$	$0.8080 \pm 0.151$	$0.7660 \pm 0.120$		0.7610

Results include the best individual model results on the test set (n=138), comparisons with state-of-the-art (SOTA) performance of deep learning rectal cancer lymph node metastasis assessment on external MRI datasets, and the average performance of radiologists on MRI calculated in a systematic review [7]. Bold values indicate the best performance for each metric in each group

**Fig. 7:**
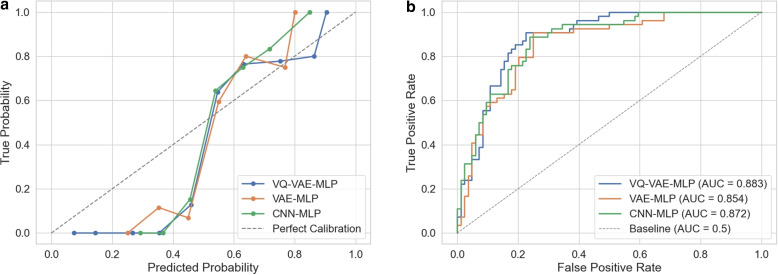
Test set calibration and receiver operating characteristic (ROC) curves for best individual VQ-VAE, VAE and CNN feature encoders with MLP classification models with a key to indicate each model type and the baseline performance (**a**, **b**)

Figure 7 shows the calibration and ROC curves for all three models on the test set, demonstrating that the models had a similar performance and calibration, with slight overfitting.

#### Comparison with prior work

Tables 4 and 7 display the results from four studies representing the current state-of-the-art for patient-level LNM staging on rectal cancer MRI using deep learning. As none of the studies reported cross-validation results and are based on individual models, they compare more directly with the best individual model results shown in Table 7.

For context, Xia et al. [30] is the most direct comparison as they used individual LN annotations and multiple instance learning. Whereas the other three studies [31–33] relied on primary tumour annotations and choosing a region of interest around them. These studies may be excluding some suspicious lymph nodes from the analysis. Additionally, only Wan et al. [31] reported a higher balanced accuracy than this study on the non-CRT group, although the proportion of patients with LNM was 19.1% and the limited test set of 86 patients had only 19 patients with positive LN pathology.

Firstly, Xia et al. [30] used a MIL framework with MRI annotations of individual LNs and a large ResNet model that incorporated LN size characteristics. The dataset included 1014 rectal cancer patients across three centres, excluding any patients that went on to receive neo-adjuvant CRT. They showed that senior radiologists could improve their balanced accuracy from 78.7% to 93.8% with model assistance. The model was deployed on an internal test cohort and two external validation cohorts, providing a good indication that the approach is generalisable.

Wan et al. [31] used a large pre-trained 3D ResNet on MRI patches with an extended field of view containing the primary tumour with some regional LNs in view, and the dataset consisted of 610 patients from the same hospital. Results were reported on a test set of 86 patients with early-stage T1-2 rectal cancer and not including any patients that received neo-adjuvant CRT. A direction comparison was done with three radiologists who had an average balanced accuracy of 54%.

Yang et al. [32] developed and compared pre-operative MRI rectal cancer lymph node metastasis staging models utilising clinical, radiomic and deep learning features. The dataset consisted of 352 patients with 286 training and 66 from an external validation cohort, all with T2 and diffusion-weighted MRI, and excluding any patients who underwent neo-adjuvant CRT treatment. Deep learning features were extracted using a pre-trained MobileNet CNN on 2D MRI images of the primary tumour, and clinical features including age, sex and radiologist-assessed N-stage, and shape, texture, first-order and wavelet features were selected by LASSO regression. A clinical validation study with a senior radiologist found they could improve their balanced accuracy from 50% to 73.9% with model assistance.

Zhao et al [33] used multiple MRI scans including axial, coronal and sagittal fields of view as well as T1, T2 and diffusion-weighted imaging, for 613 rectal cancer patients who did not have neo-adjuvant CRT treatment across 4 medical centres, including 372 training, 106 validation, 88 internal testing from one centre, and 47 external validation patients across 3 other centres. The model used 2D tumour region of interest across all MRI scans and deep learning features were extracted using a pre-trained EfficientNet, the features across all scans were then fused using an MLP for the final diagnosis. The study compared against two expert radiologists who had an average balanced accuracy of 60.4%.

#### Error analysis

To provide some analysis of difficult cases and uncertainty, test set predictions were accumulated from 208 VQ-VAE model states saved across the hyperparameter runs when the performance for one epoch was above 0.79 balanced accuracy, including multiple states from the same run.

A total of 17 N1+ patients (31.5%) were never incorrectly staged as N0, and 29 N0 patients (34.5%) were never incorrectly staged as N1+. The maximum prediction in the final individual VQ-VAE model was 0.91 and the minimum was 0.05, with 21 (15%) predictions greater than 0.75 or less than 0.25, suggesting that some cases were more certain. Average prediction confidence statistics show that 37 (27%) patients had an average confidence between 0.45 to 0.55, suggesting that the model was more uncertain on these cases and they were the most difficult to stage. Although the average prediction was correct for 23 (62%) of these cases, assuming a threshold of 0.5. In clinical applications, an expert review of these cases would be recommended.

The absolute differences in LNM probabilities between the best VQ-VAE model and the average confidence across the 208 model states had a mean of 0.049, with a standard deviation of 0.048 and range of 0.236. These statistics indicate that the model predictions were relatively certain and consistent across different hyperparameters. Across the model states, 7 N1+ patients had an average prediction of lower than 0.5 (false negative (FN)), and 22 N0 patients had an average prediction above 0.5 (false positive (FP)). Upon inspection of specific examples, a consultant radiologist confirmed they were difficult to stage with reasons including: no visible suspicious LNs or only small and medium sized suspicious LNs with an irregular border and/or heterogeneous texture in FN cases, and large benign LNs in FP cases.

Frequent FN cases had less visible LNs on average than the full set of positive cases, with 8.43 compared to 9.15. The maximum short-axis diameter LN was 10.47*mm* with an average diameter of 4.03*mm* across frequent FNs, compared to the maximum across all positive cases in the test set of 28.84*mm* and average of 4.71*mm*. Frequent false positive (FP) cases had more LNs on average than all negative cases with 8.68 compared to 6.95, and an average short-axis diameter of 4.43*mm* compared to 3.75*mm*. Additionally, 32% of the frequent FP cases had a large LN with a short-axis diameter larger than 9*mm*, which are always deemed suspicious by the clinical staging criteria [9], and 73% had a LN larger than 7*mm*.

### Clinical validation study

Table 8 displays the results of the clinical validation study, comparing the model with three radiologists including a consultant, with all results evaluated against the ground truth pathology. All three radiologists were shown the model predictions and individual LN scores after submitting the initial staging for each case however they did not change their staging for any of the scans on this validation set. Initial feedback from the radiologists was that they would have needed to use the model for a while to gain an understanding of where the model performs well and to build trust.

**Table 8:** Clinical validation study of the model across 50 cases

Group	Reader	Sensitivity	Specificity	Accuracy	F1	BA
Combined	VQ-VAE	0.9048	0.6207	0.7400	0.7451	0.7627
	Consultant Radiologist 1	0.7143	0.5517	0.6200	0.6122	0.6330
	Radiologist 2	0.9048	0.3793	0.6000	0.6552	0.6420
	Radiologist 3	0.7143	0.3448	0.5000	0.5455	0.5296
	Majority Vote	0.7619	0.3793	0.5400	0.5818	0.5706
CRT	VQ-VAE	1.000	0.4286	0.7143	0.7778	0.7142
	Consultant Radiologist 1	0.5714	0.8571	0.7143	0.6667	0.7143
	Radiologist 2	0.7143	0.4286	0.5714	0.6250	0.5714
	Radiologist 3	0.5714	0.4286	0.5000	0.5333	0.5000
	Majority Vote	0.5714	0.4286	0.5000	0.5333	0.5000
non-CRT	VQ-VAE	0.8571	0.6818	0.7500	0.7273	0.7695
	Consultant Radiologist 1	0.7857	0.4545	0.5833	0.5946	0.6201
	Radiologist 2	1.000	0.3636	0.6111	0.6667	0.6818
	Radiologist 3	0.7857	0.3182	0.5000	0.5500	0.5519
	Majority Vote	0.8571	0.3636	0.5556	0.6000	0.6104

The results demonstrate that the model had at least 12% higher balanced accuracy than the radiologists overall, and a higher F1 score on both the CRT and non-CRT patient groups. All three radiologist’s sensitivity was much lower in the CRT group compared to the non-CRT group, this decline in sensitivity following CRT reflects the known impact of treatment related changes such as fibrosis and LN shrinkage on image interpretation [4].

The confusion matrices of the three radiologists and the majority vote of all three is shown in Table 9. The model and radiologists had an average 70% agreement on staging, and there was an average of 11.67 cases where the model was correct and the radiologist was incorrect (23.3%), and 3.33 cases where the radiologist was correct and the model was incorrect (6.7%). Additionally, for the positive cases only, the model correctly called an average of 3.67 cases (17.5%) that the radiologist missed, and the radiologists correctly called an average of 1 case (4.8%) that the model missed.

**Table 9:** Confusion matrices comparing the model with three radiologists (R1 to R3) and the majority vote based on an agreement between at least two of the radiologists

	R1		R2		R3		Majority Vote	
	Correct	Incorrect	Correct	Incorrect	Correct	Incorrect	Correct	Incorrect
Model Correct	26	11	27	10	23	14	25	12
Model Incorrect	5	8	3	10	2	11	2	11

The exact McNemar test was used to compare the performance of each radiologist and the model, finding that the model did not perform significantly better than the Consultant Radiologist 1 (R1) ($p=0.20$) and Radiologist 2 (R2) ($p=0.09$), but did perform significantly better than Radiologist 3 (R3) ($p=0.004$, odds ratio 0.143 [95% CI : 0.016-0.622]) and the majority vote of all three radiologists ($p=0.01$, odds ratio 0.167 [95% CI : 0.018-0.749]). Additionally, Cohen’s kappa statistics reveal that the model had moderate agreement with the radiologists (R1: 0.344, R2: 0.425, R3: 0.319), while the agreement between radiologists was substantial (R1–R2: 0.533, R1–R3: 0.582, R2–R3: 0.565). Importantly, the model agreement with the pathology (0.496) was much higher than between individual radiologists and the pathology (R1: 0.254, R2: 0.267, R3: 0.054).

Lastly, a generalised linear mixed model was fitted with random intercepts for the radiologist reader and scan to account for the variability in radiologist skill and scan difficulty, and a fixed effect for whether the prediction was from a radiologist or the AI model. The intercept was estimated as $\beta _0 = 0.864$, the fixed effect for whether AI was used was estimated as $\beta _1=1.703$ [95% CI: 0.887-2.52], and the standard deviations of the random effects were estimated as $\sigma _{\text {radiologist}} =0$ and $\sigma _{\text {scan}} =3.29$. The results showed that relying on the AI model significantly improved predictive performance compared to radiologist predictions ($p<0.001$), with an odds ratio of 5.49 [95% CI: 2.43-12.43], indicating that after adjusting for reader and scan variability, the odds of the AI model prediction being correct were over 5 times higher than the odds of the radiologist prediction being correct. This analysis was conducted without performing a statistical power calculation, and so the results should be interpreted with caution.

Overall, this clinical validation study has provided strong evidence that the AI model performed at a level exceeding radiologists, indicating its potential as a reliable staging tool to support and augment the performance of radiologists.

### Interpretability

**Fig. 8:**
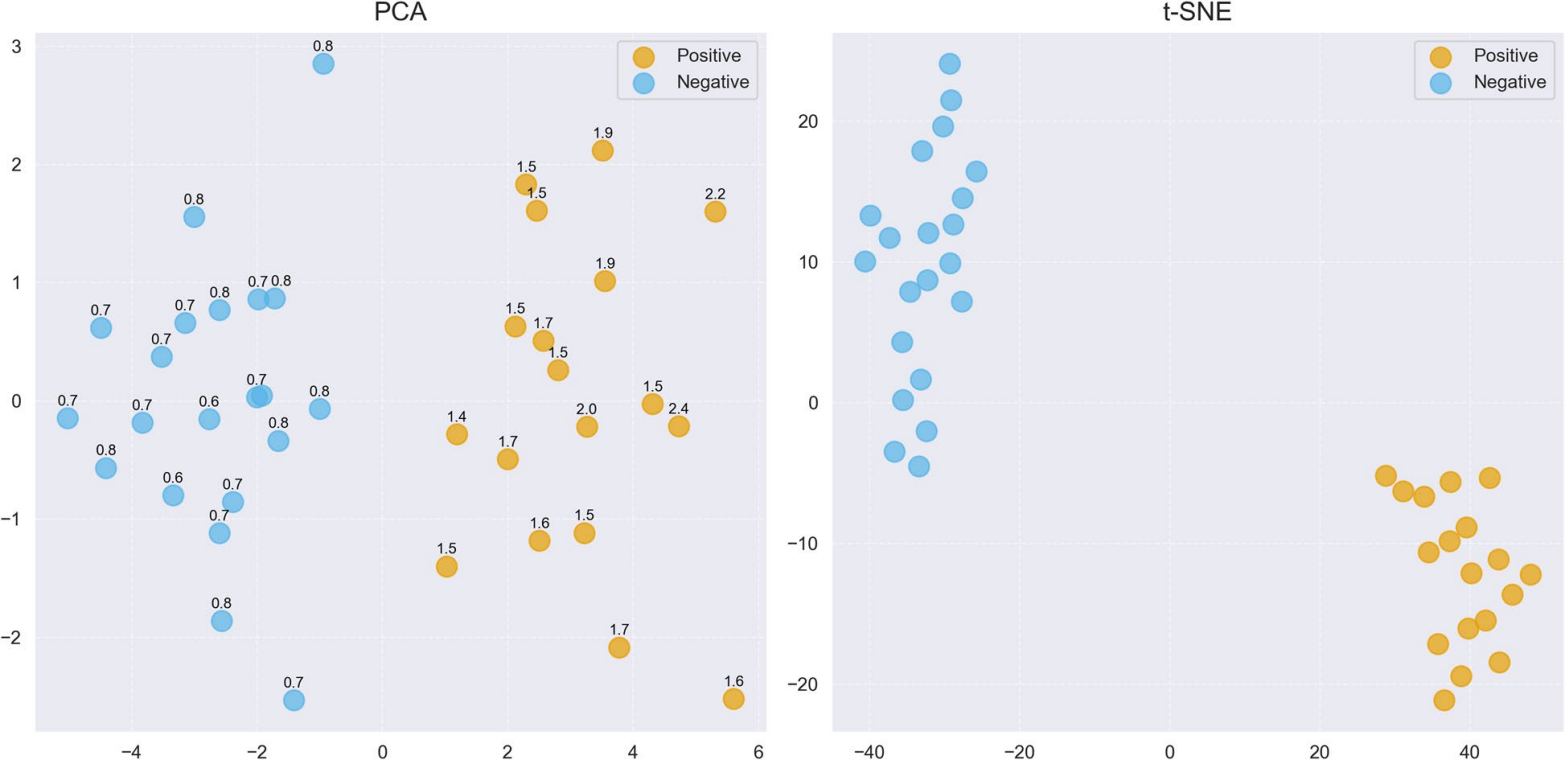
Projections of class-specific VQ-VAE codebook features, labelled with odds ratio of feature presentation in suspicious lymph nodes versus all lymph nodes

Figure 8 shows 2D projections of VQ-VAE latent vectors that were found to be significantly associated with suspicious or benign lymph nodes. The left side shows a linear projection of the features with odds ratios above each point showing the increased/decreased likelihood (compared to 1.0) that a feature appears in a suspicious LN compared to all LNs.

Table 10 displays statistics that interpret what each VQ-VAE feature code is associated with, using the average across images with the code in the centre and compared to full dataset averages. Individual LN probability averages were worked out from the test set only.

**Table 10:** Statistics on top 5 VQ-VAE features related to malignancy based on odds ratio, with percentage differences of statistics on N-stage pathology, individual LN probability, size (short-axis diameter), border irregularity (FFT) and heterogeneous signal (CV), in LN patches containing the feature code compared to the averages across the dataset

Feature code	Pathology	LN probability	Short-axis	FFT	CV	Total frequency	Odds ratio	*p*-value
141	12.5%	46.8%	66.3%	66.5%	34.2%	398	2.4	$3.5 \cdot 10^{-7}$
165	24.1%	20.2%	35.4%	23.4%	41.4%	300	2.2	$1.3 \cdot 10^{-4}$
186	16.4%	21.7%	53.9%	47.8%	10.5%	906	2.0	$1.3 \cdot 10^{-8}$
117	14.4%	22.8%	41.8%	34.1%	17.5%	1130	1.9	$5.5 \cdot 10^{-10}$
111	-0.3%	24.5%	50.7%	31.9%	30.6%	484	1.9	$2.9 \cdot 10^{-4}$

Both projections in Fig. 8 show that the malignant and benign associated codebook features were separable in latent space and formed distinct clusters, and the statistics from Table 10 provide further evidence that the VQ-VAE learned discriminative representations that capture relevant morphological patterns from the lymph nodes. Overall, these results suggest that the VQ-VAE representations were somewhat interpretable, class-specific, and corresponded to meaningful differences in lymph node morphology.

**Fig. 9:**
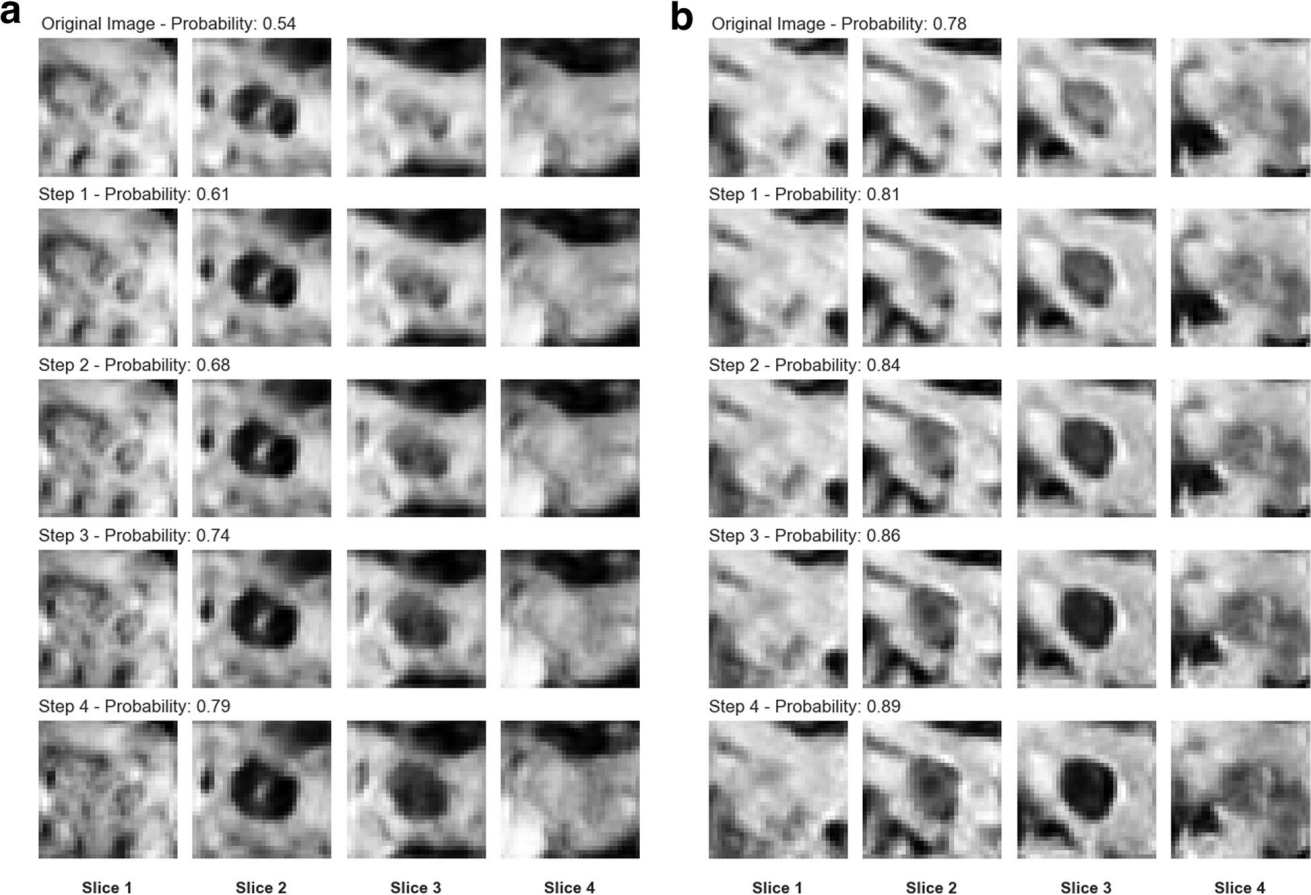
Lymph node high malignant probability direction, with the original image on the top row and reconstructed images after applying multiples of the direction vector below with displayed probability

Figure 9 shows two examples that apply the direction vector between the centroid of low probability LNs ($p<0.33$ and negative pathology) and high probability LNs ($p>0.66$ and positive pathology) in the VAE latent space. There is a smooth evolution of LN growth, increase in signal heterogeneity and border irregularity, whilst the background is fixed, demonstrating that the latent space has generalised and disentangled malignant characteristics of lymph nodes on MRI. Probability scores are displayed for each step, showing that the direction is consistent with what the classification model interprets as malignant features.

## Discussion

This study is the first to develop a deep learning model for rectal cancer lymph node metastasis staging using a broadly inclusive cohort, including patients with or without neo-adjuvant radiotherapy or chemoradiotherapy (CRT) before surgery. Restaging the lymph nodes on MRI following CRT treatment is a recognised challenge as lymph nodes often shrink, sometimes to the point of disappearing, or become obscured by fibrotic tissue [4]. In a recent study, two radiologists with 6 and 22 years of experience independently staged 136 post-treatment MRI scans and then discussed individual cases to reach a consensus. Compared to the pathology, the study reported a sensitivity of 59.6% and specificity of 65.2% [34]. For patients not receiving CRT treatment, a systematic review found that in studies using a consensus between radiologists, the average sensitivity and specificity was 73% and 75% [7]. These results compare to the cross-validated metrics of 94.8% sensitivity and 46.7% specificity for CRT, and 82.4% sensitivity and 76.9% specificity non-CRT, meaning an 8.4% and 5.6% increase in balanced accuracy compared to radiologist consensus N staging for CRT and non-CRT patient groups respectively. For the CRT group, the model had much higher sensitivity and lower specificity, which reflects the trade-off between the two metrics. Reported results used a decision boundary of 0.5, which may be adjusted to prioritise either metric depending on clinical requirements. Further work in health economics is required to set these thresholds optimally.

In addition to MRI, rectal cancer lymph node metastasis is also commonly assessed by other imaging modalities, including endoscopic ultrasound (EUS) and computed tomography (CT), which have been shown to have comparable accuracy when assessed by radiologists with sensitivity and specificity of 77% and 76% for MRI, 57% and 80% for EUS and 79% and 76% for CT [35]. Although MRI is considered the gold standard for lymph node staging by international guidelines as it has the highest contrast resolution of the soft tissues, allowing the best depiction of the lymph nodes and their anatomical surroundings out of these imaging modalities [3]. Aside from clinical assessment, AI-based techniques have been extensively applied to imaging data with both imaging-derived radiomics and deep learning features, alongside omics and other biomarker-driven approaches, to improve the prediction of lymph node metastasis [8, 36]. For example, molecular and clinicopathological biomarkers have been investigated this task, with support vector machine (SVM) models achieving a 68.3% sensitivity and 81.1% specificity [37].

The proposed radiological staging model could directly influence treatment selection and contribute to improved patient outcomes. Patients with lymph node metastasis detected on pre-treatment staging or with advanced tumours (T3-4) are generally recommended to have a locally advanced treatment regimen with CRT treatment followed by total mesorectal excision (TME) surgery [6]. In the case of early tumours (T1-2), the lymph node stage can directly influence the treatment pathway as patients with no evidence of lymph node involvement are not recommended to have neo-adjuvant CRT and could be selected for minimally invasive surgery such as local excision of the primary tumour [6]. Therefore, increased sensitivity and specificity of lymph node staging could reduce under- and over-treatment of patients.

Across the world, many clinical workflows do not have the benefit of multiple radiologist consensus, and the clinical validation study in this work is a reflection of that. In the validation sample, the model was correctly identifying more positive cases than the radiologist. The radiologist’s feedback on the model was that the individual lymph node and patient-level binary N staging probability scores were informative, and that the cases where the model was uncertain (probability close to 0.5) were broadly aligned with when they were also unsure. They also highlighted that the model was particularly valuable in the case where the model predicted positive, and they had initially thought it was radiologically negative. These cases would prompt them to re-evaluate the scans carefully, and in a real-world scenario, they would discuss the case with colleagues. The developed model is intended as a decision support tool to be used by radiologists after initial staging, to avoid biasing their findings. The tool would highlight all the detected nodes alongside confidence scores to flag potentially missed malignant nodes and help enhance diagnostic accuracy and confidence.

The Variational Autoencoder (VAE) models used in this study are well suited to the task as they can learn compact, self-supervised latent representations from small 3D MRI lymph node patches, avoiding the need for large pre-trained architectures that are poorly matched to the data size and would require inefficient upsampling or padding. By encoding the LNs into a low-dimensional space (64 for the VQ-VAE), it produces efficient and morphologically informed representations that can be transferred for classification purposes. Additionally, the presented results provide evidence that the feature representations encode class-specific and visual information, some of which aligns with clinically known diagnostic criteria, and can generalise important biological mechanisms such as malignant growth of lymph nodes.

However, some limitations and considerations for future work are covered here. Firstly, coronal scans instead of axial scans were used in some cases where suspicious lymph nodes were only visible on the coronal field of view. Radiologists typically view axial, coronal and sagittal scans, which provide complementary information when staging. Future studies would benefit from combining information from the different fields of view as well as different sequences such as T1 and diffusion-weighted MRI. This study focused on T2 axial to simplify the model as it would have required a reliable method to match lymph nodes across different MRI sequences to avoid bias from treating multiple views of the same lymph node as independent samples. The MRI slice thickness was standardised to 3.3*mm*, and given that the average LN size in this dataset was 5.5*mm*, many LNs appeared on only one or two slices. More recent scans may have thinner slices and so future studies may consider resampling older scans or applying a later cut-off date. The annotations were completed by a non-specialist, potentially leading to some LNs being omitted and other non-LN structures being included. However, 34 scan annotations were validated by a consultant GI radiologist who confirmed the annotations were of sufficient quality. Also, the N-stage pathology was used to evaluate the predictions, and so a high classification performance indicates that the annotations were accurate. The use of lower quality annotations in this task may be beneficial for future work to reduce the study development costs of using large datasets. It is important to note that matching LNs detected on MRI with the pathologic status of individual LNs is not feasible in retrospective studies due to the difficulty of matching them post-surgery. Only two previous studies have attempted this, with an obvious selection bias as they only matched the pathology of the largest detected LNs [38, 39]. The multiple instance learning approach used in this study does not rely on specific lymph node pathology and therefore may be the most feasible approach for large-scale studies.

This study was conducted at a single centre, and currently no public datasets exist for external benchmarking. Future work should prioritise external validation to evaluate the model’s generalisability across different institutions, MRI scanners and imaging protocols. A benchmark dataset would be of substantial value to the field, enabling more robust and meaningful comparisons between existing methodologies. Another consideration for future work is to use the distance from the primary tumour as a variable in the model. To enhance model explainability, future work could explore the use of the VAE latent space to provide prototype-based or counterfactual explanations, potentially offering interpretable insights into the model’s decision making process. Additionally, using a clinician-in-the-loop approach where the lymph node probabilities could be edited by radiologist based on their understanding, and then providing an updated model staging.

## Conclusion

This study has presented an end-to-end clinical decision support model for pre-operative staging of rectal cancer lymph node metastasis using MRI and clinical data. The VQ-VAE-based approach achieved the best performance with cross-validated metrics of 86.6% sensitivity and 72.4% specificity, outperforming other models and expert radiologists.

In conclusion, this study covers 12 years of clinical practice at a UK hospital where 102 patients (22.3%) had lymph node metastasis but did not undergo neo-adjuvant radiotherapy or chemoradiotherapy. The cross-validated non-CRT sensitivity suggests the model could have identified up to 84 of these cases. Additionally, the clinical validation study showed that the model identified 17.5% of patients with malignant lymph nodes that were not identified by radiologists. With approximately 10,000 cases per year in the UK [40] and 40% with lymph node metastasis pathology, clinical use could translate to an additional 700 patients per year receiving vital treatment, potentially leading to significant improvements in survival outcomes. This study provides strong evidence that a pre-operative radiological staging tool for rectal cancer lymph node metastasis is feasible and would be beneficial in clinical practice.

## Data Availability

No data are available as access to the patient data is restricted under the terms of the ethics approval.

## References

[1] Morgan E, Arnold M, Gini A, Lorenzoni V, Cabasag CJ, Laversanne M, et al. Global burden of colorectal cancer in 2020 and 2040: incidence and mortality estimates from GLOBOCAN. Gut. 2023;72(2):338–44. 10.1136/gutjnl-2022-327736. 36604116 10.1136/gutjnl-2022-327736

[2] Digital N. Cancer survival in England, cancers diagnosed 2016 to 2020, followed up to 2021. NHS Digital. 2023. https://digital.nhs.uk/data-and-information/publications/statistical/cancer-survival-in-england/cancers-diagnosed-2016-to-2020-followed-up-to-2021/. Accessed 15 May 2024.

[3] Borgheresi A, De Muzio F, Agostini A, Ottaviani L, Bruno A, Granata V, et al. Lymph Nodes Evaluation in Rectal Cancer: Where Do We Stand and Future Perspective. J Clin Med. 2022;11(9):1–26. 10.3390/jcm11092599. 10.3390/jcm11092599 PMC9104021 35566723

[4] Horvat N, Carlos Tavares Rocha C, Clemente Oliveira B, Petkovska I, Gollub MJ. MRI of Rectal Cancer: Tumor Staging, Imaging Techniques, and Management. RadioGraphics. 2019;39(2):367–87. PMID: 30768361. 10.1148/rg.2019180114. 10.1148/rg.2019180114 PMC6438362 30768361

[5] Isensee F, Jaeger PF, Kohl SAA, Petersen J, Maier-Hein KH. nnU-Net: a self-configuring method for deep learning-based biomedical image segmentation. Nat Methods. 2021;18(2):203–11. 10.1038/s41592-020-01008-z. 33288961 10.1038/s41592-020-01008-z

[6] Colorectal cancer: Diagnosis and management. National Institute for Health and Care Excellence (NICE). 2020. https://www.nice.org.uk/guidance/ng151/chapter/Recommendations#management-of-local-disease. Accessed 17 Jun 2025.

[7] Zhuang Z, Zhang Y, Wei M, Yang X, Wang Z. Magnetic Resonance Imaging Evaluation of the Accuracy of Various Lymph Node Staging Criteria in Rectal Cancer: A Systematic Review and Meta-Analysis. Front Oncol. 2021;11(July). 10.3389/fonc.2021.709070. 10.3389/fonc.2021.709070 PMC8315047 34327144

[8] Keel B, Quyn A, Jayne D, Relton SD. State-of-the-art performance of deep learning methods for pre-operative radiologic staging of colorectal cancer lymph node metastasis: a scoping review. BMJ Open. 2024;14(12). 10.1136/bmjopen-2024-086896. 10.1136/bmjopen-2024-086896 PMC11624802 39622569

[9] Beets-Tan RGH, Lambregts DMJ, Maas M, Bipat S, Barbaro B, Curvo-Semedo L, et al. Magnetic resonance imaging for clinical management of rectal cancer: Updated recommendations from the 2016 European Society of Gastrointestinal and Abdominal Radiology (ESGAR) consensus meeting. Eur Radiol. 2018;28(4):1465–75. 10.1007/s00330-017-5026-2. 29043428 10.1007/s00330-017-5026-2 PMC5834554

[10] Pérez-García F, Sparks R, Ourselin S. TorchIO: a Python library for efficient loading, preprocessing, augmentation and patch-based sampling of medical images in deep learning. Comput Methods Programs Biomed. 2021;106236. 10.1016/j.cmpb.2021.106236. 10.1016/j.cmpb.2021.106236 PMC8542803 34311413

[11] Isensee F, Wald T, Ulrich C, Baumgartner M, Roy S, Maier-Hein K, et al. nnU-Net Revisited: A Call for Rigorous Validation in 3D Medical Image Segmentation. In: proceedings of Medical Image Computing and Computer Assisted Intervention – MICCAI 2024. vol. LNCS 15009. Springer Nature Switzerland. 2024. 10.1007/978-3-031-72114-4_47.

[12] Fedorov A, Beichel R, Kalpathy-Cramer J, Finet J, Fillion-Robin JC, Pujol S, et al. 3D Slicer as an Image Computing Platform for the Quantitative Imaging Network. Magn Reson Imaging. 2012;30(9):1323–41. 10.1016/j.mri.2012.05.001. 22770690 10.1016/j.mri.2012.05.001 PMC3466397

[13] 3D Slicer: An Open Source Software Platform for Medical Image Computing and Research. 2024. https://www.slicer.org/. Accessed 24 Oct 2024.

[14] Lecun Y, Bengio Y, Hinton G. Deep learning. Nature. 2015;521(7553):436–44. 10.1038/nature14539. 26017442 10.1038/nature14539

[15] Chen C, Mat Isa NA, Liu X. A review of convolutional neural network based methods for medical image classification. Comput Biol Med. 2025;185:109507. 10.1016/j.compbiomed.2024.109507. 39631108 10.1016/j.compbiomed.2024.109507

[16] Kingma DP, Welling M. Auto-Encoding Variational Bayes. arXiv. 2013. 10.48550/ARXIV.1312.6114.

[17] Sreelekshmi V, Pavithran K, Nair JJ. Unleashing the Power of Hierarchical Variational Autoencoder for Predicting Breast Cancer. IEEE Access. 2024;12:195658–70. 10.1109/ACCESS.2024.3518612.

[18] Keel B, Quyn A, Jayne D, Relton SD. Variational Autoencoders for Feature Exploration and Malignancy Prediction of Lung Lesions. In: British Machine Vision Conference (BMVC). 2023. 10.48550/arXiv.2311.15719.

[19] van den Oord A, Vinyals O, Kavukcuoglu K. Neural discrete representation learning. In: Proceedings of the 31st International Conference on Neural Information Processing Systems, NeurlIPS 2017. NIPS’17. Red Hook: Curran Associates Inc.; 2017. pp. 6309–18. 10.48550/arXiv.1711.00937.

[20] Wang Z, Bovik AC, Sheikh H, Simoncelli E. Image quality assessment: from error visibility to structural similarity. IEEE Trans Image Process. 2004;13(4):600–12. 10.1109/TIP.2003.819861. 15376593 10.1109/tip.2003.819861

[21] Zhang R, Isola P, Efros AA, Shechtman E, Wang O. The Unreasonable Effectiveness of Deep Features as a Perceptual Metric. In: 2018 IEEE/CVF Conference on Computer Vision and Pattern Recognition. 2018. pp. 586–95. 10.1109/CVPR.2018.00068.

[22] Hendrycks D, Gimpel K. Gaussian Error Linear Units (GELUs). arXiv. 2016. 10.48550/arXiv.1606.08415.

[23] Taylor CC, Millien VO, Hou JK, Massarweh NN. Association Between Inflammatory Bowel Disease and Colorectal Cancer Stage of Disease and Survival. J Surg Res. 2020;247:77–85. 10.1016/j.jss.2019.10.040. 31767275 10.1016/j.jss.2019.10.040

[24] Kyrgiou M, Kalliala I, Markozannes G, Gunter MJ, Paraskevaidis E, Gabra H, et al. Adiposity and cancer at major anatomical sites: umbrella review of the literature. BMJ. 2017;356. 10.1136/bmj.j477. 10.1136/bmj.j477 PMC5421437 28246088

[25] Zhang H, Zhang C, Zheng Z, Ye F, Liu Y, Zou S, et al. Chemical shift effect predicting lymph node status in rectal cancer using high-resolution MR imaging with node-for-node matched histopathological validation. Eur Radiol. 2017;27(9):3845–55. 10.1007/s00330-017-4738-7. 28168369 10.1007/s00330-017-4738-7

[26] Cardoso MJ, Li W, Brown R, Ma N, Kerfoot E, Wang Y, et al. MONAI: An open-source framework for deep learning in healthcare. 2022. 10.48550/arXiv.2211.02701.

[27] Biewald L. Experiment Tracking with Weights and Biases. 2020. https://www.wandb.com/. Accessed 2 Jul 2024.

[28] Lin TY, Goyal P, Girshick R, He K, Dollar P. Focal Loss for Dense Object Detection. IEEE Trans Pattern Anal Mach Intell. 2018;PP:1. 10.1109/TPAMI.2018.2858826. 10.1109/TPAMI.2018.2858826 30040631

[29] van der Maaten L, Hinton G. Visualizing Data using t-SNE. J Mach Learn Res. 2008;9(86):2579–605.

[30] Xia W, Li D, He W, Pickhardt PJ, Jian J, Zhang R, et al. Multicenter Evaluation of a Weakly Supervised Deep Learning Model for Lymph Node Diagnosis in Rectal Cancer at MRI. Radiol Artif Intell. 2024;6(2). 10.1148/ryai.230152. 10.1148/ryai.230152 PMC10982819 38353633

[31] Wan L, Hu J, Chen S, Zhao R, Peng W, Liu Y, et al. Prediction of lymph node metastasis in stage T1–2 rectal cancers with MRI-based deep learning. Eur Radiol. 2023;3638–46. 10.1007/s00330-023-09450-1. 10.1007/s00330-023-09450-1 36905470

[32] Yang Y, Han K, Xu Z, Cai Z, Zhao H, Hong J, et al. Development and Validation of Multiparametric MRI-based Interpretable Deep Learning Radiomics Fusion Model for Predicting Lymph Node Metastasis and Prognosis in Rectal Cancer: A Two-center Study. Acad Radiol. 2025;32(5):2642–54. 10.1016/j.acra.2024.11.045. 39638641 10.1016/j.acra.2024.11.045

[33] Zhao J, Zheng P, Xu T, Feng Q, Liu S, Hao Y, et al. Preoperative Assessment of Lymph Node Metastasis in Rectal Cancer Using Deep Learning: Investigating the Utility of Various MRI Sequences. Ann Surg Oncol. 2025;(0123456789). 10.1245/s10434-025-17717-8. 10.1245/s10434-025-17717-8 40553356

[34] Zhang Z, Chen Y, Wen Z, Wu X, Que Y, Ma Y, et al. MRI for nodal restaging after neoadjuvant therapy in rectal cancer with histopathologic comparison. Cancer Imaging. 2023;23(1):1–8. 10.1186/s40644-023-00589-0. 37443085 10.1186/s40644-023-00589-0 PMC10339540

[35] Li XT, Sun YS, Tang L, Cao K, Zhang XY. Evaluating local lymph node metastasis with magnetic resonance imaging, endoluminal ultrasound and computed tomography in rectal cancer: a meta-analysis. Colorectal Dis. 2015;17(6):O129–35. 10.1111/codi.12909. 25628186 10.1111/codi.12909

[36] Peng W, Qiao H, Mo L, Guo Y. Progress in the diagnosis of lymph node metastasis in rectal cancer: a review. Front Oncol. 2023;Volume 13 - 2023. 10.3389/fonc.2023.1167289. 10.3389/fonc.2023.1167289 PMC10374255 37519802

[37] Fan Xj, Wan Xb, Huang Y, Cai Hm, Fu Xh, Yang Zl, et al. Epithelial – mesenchymal transition biomarkers and support vector machine guided model in preoperatively predicting regional lymph node metastasis for rectal cancer. Brit J Cancer. 2012;1735–41. 10.1038/bjc.2012.82. 10.1038/bjc.2012.82 PMC3364123 22538975

[38] Li J, Zhou Y, Wang P, Zhao H, Wang X, Tang N, et al. Deep transfer learning based on magnetic resonance imaging can improve the diagnosis of lymph node metastasis in patients with rectal cancer. Quant Imaging Med Surg. 2021;11(6):2477–85. 10.21037/qims-20-525. 34079717 10.21037/qims-20-525 PMC8107313

[39] Ozaki K, Kurose Y, Kawai K, Kobayashi H, Itabashi M, Hashiguchi Y, et al. Development of a Diagnostic Artificial Intelligence Tool for Lateral Lymph Node Metastasis in Advanced Rectal Cancer. Dis Colon Rectum. 2023;66(12):E1246–53. 10.1097/DCR.0000000000002719. 37260284 10.1097/DCR.0000000000002719

[40] State of the Nation Report. National Bowel Cancer Audit (NBOCA). 2025. https://www.nboca.org.uk/wp-content/uploads/2025/01/NBOCA-State-of-the-Nation-Report-2024_v1.0_09.01.2025.pdf. Accessed 22 Aug 2025.

